# Supplementary material for: Node-layer duality in networked systems
Source: Nat Commun. 2024 Jul 18;15:6038. doi: 10.1038/s41467-024-50176-5 (PMC11255284; doi:10.1038/s41467-024-50176-5)
Supplement: Supplementary file 1 — Supplementary Information [file 41467_2024_50176_MOESM1_ESM.pdf]

Supplementary Text

Node-layer duality in networked systems

Charley Presigny[1], Marie-Constance Corsi[1], Fabrizio De Vico Fallani[1,*]

1 Sorbonne Université, Institut du Cerveau - Paris Brain Institute - ICM, CNRS, Inria, Inserm, AP-HP, Hôpital de la Pitié Salpêtrière, F-75013, Paris, France

* corresponding author: fabrizio.de-vico-fallani@inria.fr

1 Stochastic multilayer rewiring model

In this section, we detail the computations of the analytical results presented in the main text. We first introduce the necessary notations, then we derive the expected layer multidegree centrality averaged over all the possible configurations of the stochastic rewiring model. The node multidegree centrality degree derivation is not shown since it follows the exact same canvas.

1.1 Notations

Latin letters encode for node indices and greek letters for layer indices in the nodewise description.

- N is the number of nodes in the nodewise description (the classical one).
- M: number of layers in the noderwise description.
- $k_{\mathcal{X}}$: node multidegree centrality vector.
- $k_{\mathcal{Y}}$: layer multidegree degree centrality vector.
- L: total number of unique edges in the system.
- L_{max}: total possible number of unique edges in the system. $L_{max} = \frac{MN(N-1)}{2}$ for multiplex networks with no self-loops.
- $\pi_{ij}^{\alpha\beta}(n)$: weight of edge between node i in layer α and node j in layer β after n rewiring. For binary networks it equals 0 or 1. $\pi_{ij}^{\alpha\beta}(0)$ is the related weights before any rewiring.
- $\Pi_{ab}^{\mu,\nu}(n)$: probability to select the edge connecting a in layer μ to node b in layer ν at the n-th rewiring.
- $\omega_{ab,\mu\nu\to ij,\alpha\beta}(n)$: transition probability to move the edge connecting a in layer μ to node b in layer ν to edge connecting i in layer α and j in layer β at the n-th rewiring .
- $p(rule = r)$: probability to select a given rewiring rule. It can take the value $r = \{node, layer, tel\}$ associated with the probabilities $p_{node}, p_{layer}, p_{tel}$ respectively. Note that $p_{node} + p_{layer} + p_{tel} = 1$.

1.2 Preliminaries and hypothesis

We make the hypothesis that *multilinks are allowed in the rewiring process i.e., edges can be rewired where edges exist already* [1]. This hypothesis ensures that the rewiring process is markovian and so, analytically tractable. In other words, it ensures that the transition probability ω does not depend on every previous rewiring step. We also impose the constraint that each edge that is rewired cannot be rewired anymore in the network. This ensures the number of rewiring n to be equal to the number of unique edges in the system L at the maximum. Furthermore, we reduce our analysis to multilayer networks which exibits the same set of nodes across each layer.

Nonetheless, real-world multilayer networks do not exihibit multilinks in general. It implies that the transition probability is zero for an edge that exists already in a realistic rewiring. Also, it implies that the transition probability to rewire an edge in any available position is comparatively higher in the analytical rewiring than in a realistic rewiring. Therefore, the higher is the *density*, the higher is the discrepancy between the model and the realistic rewiring. Since real-world networks are sparse (low density), the discrepancy should stay low in practice. Indeed, we show that the model holds for sparse full multilayer networks and sparse multiplex networks (**Fig S2**).

Our objective is to find $\overline{\pi_{ij}^{\alpha\beta}(n)}$, the value of edge connecting (i,α) to (j, β) averaged over all possible configurations of the rewiring model after n rewiring. If we denote $P(\pi_{ij}^{\alpha\beta}(n) = x)$, the probability for the latter edge to have the value x at the n-th rewiring, we can write $\overline{\pi_{ij}^{\alpha\beta}(n)} = \sum_x xP(\pi_{ij}^{\alpha\beta}(n) = x)$. Since we deal with undirected binary networks (edges have value 0 or 1) we obtain that $\overline{\pi_{ij}^{\alpha\beta}(n)} = P(\pi_{ij}^{\alpha\beta}(n) = 1)$. Since we are ultimately interested in the multidegree centrality vectors, we will progressively integrate $\overline{\pi_{ij}^{\alpha\beta}(n)}$ in the following derivation.

1.3 $\Pi(n)$ does not depend on the rewiring step

By construction, each edge that is rewired at step n cannot be rewired anymore at subsequent steps. Also, it means that the population of edge to be rewired progressively decreases as the network is rewired. Furthermore, $\Pi_{ab}^{\mu\nu}(n)$ i.e, the probability of selecting the edge connecting (a,μ) and (b, ν) at the n-th rewiring depends only on what happens on the $(n-1)$th rewiring step: either the edge was selected or it was not selected at a previous step and so the probability of selecting an edge is zero for all subsequent steps. Let us denote the conditional probability $\Pi_{ab}^{\mu\nu}(n|n-1)$ for any edge to be selected at timestep n knowing it was not at step $n-1$. It implies that:

$$\Pi_{ab}^{\mu\nu}(n) = \Pi_{ab}^{\mu\nu}(n|n-1) \times \overline{\Pi_{ab}^{\mu\nu}(n-1|n-2)} \times .... \times \overline{\Pi_{ab}^{\mu\nu}(2|1)\Pi_{ab}^{\mu\nu}(1)}, \tag{1}$$

where bars, represent the complementary probability. At any timestep n, the edge is drawn uniformly at random among the population of edges that is still rewirable. If we suppose that the edge connecting (a, μ) to (b, ν) is not rewired at time $n-1$, it implies that:

$$\begin{aligned}\overline{\Pi_{ab}^{\mu\nu}(n|n-1)} &= \pi_{ab}^{\mu\nu}(0)\left(1 - \frac{1}{L-(n-1)}\right)\\ &= \pi_{ab}^{\mu\nu}(0)\frac{L-n}{L-n+1},\end{aligned} \tag{2}$$

where $\pi_{ab}^{\mu\nu}(0)$ ensures that the binary edge is present in the initial network before any rewiring. Since it is binary, we make it appear only once in related expressions. Using

Eqs. 1,2, read:

$$\Pi_{ab}^{\mu\nu}(n) = \frac{\pi_{ab}^{\mu\nu}(0)}{L-(n-1)} \prod_{i=1}^{n-1} \frac{L-i}{L-i+1}$$
$$= \frac{\pi_{ab}^{\mu\nu}(0)}{L-(n-1)} \frac{L-(n-1)}{L}$$
$$= \frac{\pi_{ab}^{\mu\nu}(0)}{L} \quad (3)$$

Therefore, the probability to select a given edge stays constant for any rewiring step.

1.4 Derivation of the expected multidegree centrality values

Following the notations, we derive the following rate equation for $\overline{\pi_{ij}^{\alpha\beta}(n+1)} = P(\pi_{ij}^{\alpha\beta}(n+1) = 1)$. Since we derive only with expected values, we lighten the notations by not considering overlines in the rest of the document (e.g. $\overline{\pi_{ij}^{\alpha\beta}(n)} = \pi_{ij}^{\alpha\beta}(n)$):

$$\pi_{ij}^{\alpha\beta}(n+1) - \pi_{ij}^{\alpha\beta}(n) = \sum_{(a\mu,b\nu)\neq(i\alpha,j\beta)} \Pi_{ab}^{\mu\nu}(n)\omega_{a\mu,b\nu\to i\alpha,j\beta} - \Pi_{ij}^{\alpha\beta}(n)\omega_{i\alpha,j\beta\to a\mu,b\nu} \quad (4)$$

Note that we forbid the edge to be rewired in the exact same place as it is. Once selected, the edge connecting (i,α) to (j,β) necessarily goes somewhere in the multilayer network i.e, $\sum_{(a\mu,b\nu)\neq(i\alpha,j\beta)} \omega_{i\alpha,j\beta\to a\mu,b\nu} = 1$. The transition probability is conditioned by the selected rewiring rule whose probability is $p(rule = r)$. Using $\sum_r p(rule = r) = 1$ the previous formula read:

$$\pi_{ij}^{\alpha\beta}(n+1) - \pi_{ij}^{\alpha\beta}(n) = \sum_r \left(\sum_{(a\mu,b\nu)\neq(i\alpha,j\beta)} \Pi_{ab}^{\mu\nu}(n)\omega_{(a\mu,b\nu\to i\alpha,j\beta|r)} - \Pi_{ij}^{\alpha\beta}(n) \right) p(rule = r) \quad (5)$$

Each rule forces the edge to be rewired in a particular way e.g, for $r = node$, the edge connecting (i,α) in (j,β) is necessarily rewired in a position that conserves the layer indices (see below). The transition probability conditioned to the rewiring rules read:

$$\omega_{(a\mu,b\nu\to i\alpha,j\beta|r)} = \begin{cases} \frac{\delta(\cdot\mu,\cdot\nu=\cdot\alpha,\cdot\beta)}{L_{max}^{\alpha\beta}-1} & \text{if } r = node \\ \frac{\delta(a\cdot,b\cdot=i\cdot,j\cdot)}{L_{max}^{ij}-1} & \text{if } r = layer \\ \frac{\delta(a\mu,b\nu)-\delta(a\cdot,b\cdot=i\cdot,j\cdot)-\delta(\cdot\mu,\cdot\nu=\cdot\alpha,\cdot\beta)}{L_{max}-L_{max}^{\alpha\beta}-L_{max}^{ij}+1} & \text{if } r = tel, \end{cases}$$

where $\delta(\cdot\mu, \cdot\nu = \cdot\alpha, \cdot\beta) = 1$, if layer indices $\mu = \alpha, \nu = \beta$ and $\delta(\cdot\mu, \cdot\nu = \cdot\alpha, \cdot\beta) =$ 0,otherwise. $\delta(a\cdot, b\cdot = i\cdot, j\cdot)$ is defined the same way for node indices $a = i, b = j$. $L_{max}^{\alpha\beta}$ is the total possible number of unique edges in the (inter)layer α, β, L_{max}^{ij} is the total possible number of unique edges for the pair i,j. Since we deal with multilayer networks composed of replica nodes, the possible number of uniques edges for a (inter)layer and for a pair of nodes does not depend on the node and layer indices. Consequently, we note $L_{max}^{ij} = L_p$ and $L_{max}^{\alpha\beta} = L_\lambda$. For convenience we also write $L_{max} - \Delta L = L_{max} - L_{max}^{\alpha\beta} - L_{max}^{ij} + 1$. By expliciting Eq. 5, using Eq. 3 and the previous ω expressions,we obtain:

$$\pi_{ij}^{\alpha\beta}(n+1)-\pi_{ij}^{\alpha\beta}(n)=\frac{p_{node}}{L}\left(\sum_{(a\mu,b\nu)\neq(i\alpha,j\beta)}\frac{\delta(\cdot\mu,\cdot\nu=\cdot\alpha,\cdot\beta)\pi_{ab}^{\mu\nu}(0)}{L_\lambda-1}-\pi_{ij}^{\alpha\beta}(0)\right) \tag{6}$$

$$+\frac{p_{layer}}{L}\left(\sum_{(a\mu,b\nu)\neq(i\alpha,j\beta)}\frac{\delta(a\cdot,b\cdot=i\cdot,j\cdot)\pi_{ab}^{\mu\nu}(0)}{L_p-1}-\pi_{ij}^{\alpha\beta}(0)\right)$$

$$+\frac{p_{tel}}{L}\left(\sum_{(a\mu,b\nu)\neq(i\alpha,j\beta)}\frac{\left(\delta(a\mu,\nu b)-\delta(a\cdot,b\cdot=i\cdot,j\cdot)-\delta(\cdot\mu,\cdot\nu=\cdot\alpha,\cdot\beta)\right)\pi_{ab}^{\mu\nu}(0)}{L_{max}-\Delta L}-\pi_{ij}^{\alpha\beta}(0)\right)$$

We define $L_{\alpha\beta}(0)$ and $L_{ij}(0)$ as the total number of unique edges present in the initial multilayer network in the (inter)layer α,β and the total number of unique edges present in the initial multilayer network in the pair *i,j*, respectively. Simplifying the previous equation leads to:

$$\pi_{ij}^{\alpha\beta}(n+1)-\pi_{ij}^{\alpha\beta}(n)=\frac{p_{node}}{L}\left(\frac{L_{\alpha\beta}(0)-L_\lambda\pi_{ij}^{\alpha\beta}(0)}{L_\lambda-1}\right) \tag{7}$$

$$+\frac{p_{layer}}{L}\left(\frac{L_{ij}(0)-L_p\pi_{ij}^{\alpha\beta}(0)}{L_p-1}\right)$$

$$+\frac{p_{tel}}{L}\left(\frac{((L-L_{\alpha\beta}(0)-L_{ij}(0))-(L_{max}-\Delta L+1)\pi_{ij}^{\alpha\beta}(0)}{\text{Ł}_{max}-\Delta L}\right)$$

We note that the right-handside is a constant that depends only on the structure of the initial multilayer network and not on the rewiring step. Therefore $\pi_{ij}^{\alpha\beta}(n)$ follows a simple arithmetic sequence that we can explicit:

$$\pi_{ij}^{\alpha\beta}(n)=\pi_{ij}^{\alpha\beta}(0)+\frac{np_{node}}{L}\left(\frac{L_{\alpha\beta}(0)-L_\lambda\pi_{ij}^{\alpha\beta}(0)}{L_\lambda-1}\right) \tag{8}$$

$$+\frac{np_{layer}}{L}\left(\frac{L_{ij}(0)-L_p\pi_{ij}^{\alpha\beta}(0)}{L_p-1}\right)$$

$$+\frac{np_{tel}}{L}\left(\frac{(L-L_{ij}(0)-L_{\alpha\beta}(0)-(L_{max}-\Delta L+1)\pi_{ij}^{\alpha\beta}(0)}{L_{max}-\Delta L}\right)$$

Since we are interested in knowing the expected node and layer multidegree centrality of a network that underwent the rewiring process, we need to sum the above expression. Here, we show the derivation for the layer multidegree centrality by summing over the node component *i,j*. Note that $\sum_{i>j}1=L_\lambda$.We set $r=\frac{n}{L}$ with $r\in[0,1]$:

$$L_{\alpha\beta}(r)=L_{\alpha\beta}(0)+rp_{node}\left(\frac{L_{\alpha\beta}(0)L_\lambda-L_{\alpha\beta}(0)L_\lambda}{L_\lambda-1}\right) \tag{9}$$

$$+rp_{layer}\left(\frac{L-L_pL_{\alpha\beta}(0)}{L_p-1}\right)$$

$$+rp_{tel}\left(\frac{(L_\lambda-1)L-L_{\alpha\beta}(0)(L_{max}-L_{p)}}{L_{max}-\Delta L}\right)$$

The term that multiplies p_{node} equals zero, which is expected by construction of the stochastic rewiring model. By summing over one of the layer component, we obtain the layer multidegree centrality:

$$k^{\alpha}_{\mathcal{Y}}(r) = k^{\alpha}_{\mathcal{Y}}(0) + rp_{layer}\left(\frac{T_{\lambda}L - L_p k^{\alpha}_{\mathcal{Y}}(0)}{L_p - 1}\right) \tag{10}$$
$$+rp_{tel}\left(\frac{T_{\lambda}(L_{\lambda}-1)L - k^{\alpha}_{\mathcal{Y}}(0)(L_{max}-L_p)}{L_{max}-\Delta L}\right),$$

where T_{λ} is a coefficient that depends on the multilayer type (multilayer or multiplex- see below). Similarly for the node multidegree centrality we obtain:

$$k^{i}_{\mathcal{X}}(r) = k^{i}_{\mathcal{X}}(0) + rp_{node}\left(\frac{T_{p}L - L_{\lambda} k^{i}_{\mathcal{X}}(0)}{L_{\lambda} - 1}\right) \tag{11}$$
$$+rp_{tel}\left(\frac{T_{p}(L_{p}-1)L - k^{i}_{\mathcal{X}}(0)(L_{max}-L_{\lambda})}{L_{max}-\Delta L}\right),$$

where T_p is a coefficient that depends on the multilayer type (multiplex, full multilayer). Note that Eqs.10, 11 are valid for any initial network topology.

Depending on the type of multilayer networks L_p, L_{λ}, T_p, T_{λ} have different values. We distinguish two main types of multilayer networks with a replica set of nodes across layers:

- Multiplex network: edges are present within layers and are only allowed between replica nodes across layers. No self-loops are included.
- Multilayer networks: links are allowed within layers, and between any nodes across layers. Self-loops are included.

	multiplex network	full multilayer network
L_p	M	M^2
L_{λ}	$\frac{N(N-1)}{2}$	$\frac{N^2}{2}$
T_p	N-1	N
T_{λ}	2	2M

T_{λ} counts the number of terms needed to go from $L_{\alpha\beta}$ to $k^{\alpha}_{\mathcal{Y}}$. Since $L_{\alpha\beta} = \sum_{i>j}\pi^{\alpha\beta}_{ij}$ and $k^{\alpha}_{\mathcal{Y}} = \sum_{ij\beta}\pi^{\alpha\beta}_{ij}$, we have that $k^{\alpha}_{\mathcal{Y}} = 2\sum_{\beta}L_{\alpha\beta}$. For multiplex networks, the sum reduces to one term so $T_{\lambda} = 2$ and for multilayer networks it reduces to M terms so $T_{\lambda} = 2M$. T_p counts the number of terms needed to go from L_{ij} to $k^{i}_{\mathcal{X}}$. Since L_{ij} is the number of unique edges from i to j, $k^{i}_{\mathcal{X}} = \sum_j L_{ij}$. For multiplex networks, the sum reduces to $N-1$ (no self-loops) term so $T_p = N-1$ and for multilayer networks it reduces to N terms so $T_p = N$.

1.5 Expression of $k_{\mathcal{Y}}(r)$ and the distances in the thermodynamic limit

In the previous section, we obtained the expression of the node and layer multidegree centralities averaged over an infinite number of realizations of the algorithm (see

Eqs.10,11). Note that those equations are valid for any N and M. In the limit of large networks ($N >> 1$ and $M >> 1$), and remembering that $L_{max} = L_p L_\lambda$ and $1 - p_{node} = p_{layer} + p_{tel}$, the previous formulae reduce to simpler, more interpretable expressions:

$$\lim_{M,N\to\infty} L_{\alpha\beta}(r) = L_{\alpha\beta}(0) + r(1-p_{node})(\frac{L}{L_p} - L_{\alpha\beta}(0))$$
$$\lim_{M,N\to\infty} k^{\alpha}_{\mathcal{Y}}(r) = k^{\alpha}_{\mathcal{Y}}(0) + r(1-p_{node})(\langle k_{\mathcal{Y}}\rangle - k^{\alpha}_{\mathcal{Y}}(0)) \tag{12}$$

where $\langle k_{\mathcal{Y}}\rangle$ is the ensemble average of the layer multidegree centrality. These findings imply that the multidegree centrality after rewiring is driven by the difference between the initial value and the ensemble average. The more the rewiring alters the connected layers (i.e. $p_{node} \to 0$), the more their multidegree centrality will be attracted by the average value, leading to more homogeneous layerwise distributions (**Fig S1**). Same conclusions can be obtained for the node multidegree centrality $k^{i}_{\mathcal{Y}}(r)$.

We finally compute the distance between the initial network and the expected layer multidegree centrality. Distances are the euclidean distances between the components of multidegree centrality vector of two multilayer networks. With the multidegree centrality formula, distance reads:

$$d(k_{\mathcal{Y}}, k_{\mathcal{Y}}(r)) = \sqrt{\sum_k \left(r(1-p_{node})(\langle k_{\mathcal{Y}}\rangle - k^{\alpha}_{\mathcal{Y}}(0))^2\right)} \tag{13}$$
$$d(k_{\mathcal{Y}}, k_{\mathcal{Y}}(r)) = r\sqrt{M}(1-p_{node})\sigma(k_{\mathcal{Y}}), \tag{14}$$

where $\sigma(k_{\mathcal{Y}})$ is the standard deviation of the layer multidegree centrality distribution. Similarly the node multidegree centrality distance read:

$$d(k_{\mathcal{X}}, k_{\mathcal{X}}(r)) = r\sqrt{N}(1-p_{layer})\sigma(k_{\mathcal{X}}), \tag{15}$$

where $\sigma(k_{\mathcal{X}})$ is the standard deviation of the node multidegree centrality distribution. Note that Eqs. 12,14,15 are valid for any initial topology of the rewired multilayer network.

1.6 Close-form of the standard deviation of random multilayer networks

We need to derive the standard deviation of the node and layer multidegree centrality for random multilayer networks to obtain a close-form of the distance. Since we deal with random multilayer networks, their number of edges follows a binomial law. Let us define L^{inter}_{max},L^{intra}_{max}, $L^{replica}_{max}$, the maximum number of interlayer, intralayer and replica edges in the network, per interacting unit $\mathcal{X}, \mathcal{Y}$ (node or layers), respectively. The probability of a unit to have L^{intra} intralayer edges, L^{inter} interlayer edges and $L^{replica}$ read:

$$P(l^{intra} = L^{intra}) = \binom{L^{intra}_{max}}{L^{intra}} q^{L^{intra}} (1-q)^{L^{intra}_{max} - L^{intra}}$$
$$P(l^{inter} = L^{inter}) = \binom{L^{inter}_{max}}{L^{inter}} q^{L^{inter}} (1-q)^{L^{inter}_{max} - L^{inter}} \tag{16}$$
$$P(l^{replica} = L^{replica}) = \binom{L^{replica}_{max}}{L^{replica}} q^{L^{replica}} (1-q)^{L^{replica}_{max} - L^{replica}},$$

where q is the probability parameter. The contribution of each edge to the multidegree centralities depends on its type.

Actually, each intralayer edge contributes to two units of the layer multidegree centrality $k_{\mathcal{Y}}^{intra} = 2L^{intra}$ and each interlayer edge and each replica edge contribute to one unit of the layer multidegree centrality $k_{\mathcal{Y}}^{inter} = L^{inter}$, $k_{\mathcal{Y}}^{replica} = L^{replica}$. Each replica edge contributes to two units of the node multidegree centrality $k_{\mathcal{X}}^{replica} = 2L^{replica}$ and intralayer and interlayer edges contribute both to one unit for the node multidegree centrality $k_{\mathcal{X}}^{intra} = L^{intra}$ and $k_{\mathcal{X}}^{inter} = L^{inter}$. The following table makes explicit L_{max}^{intra}, L_{max}^{inter} and $L_{max}^{replica}$:

	L_{max}^{intra}	L_{max}^{inter}	$L_{max}^{replica}$
$\mathcal{X}$	$M(N-1)$	$\frac{2M(M-1)(N-1)}{2}$	$\frac{M(M-1)}{2}$
$\mathcal{Y}$	$\frac{N(N-1)}{2}$	$\frac{2N(N-1)(M-1)}{2}$	$N(M-1)$

Since the number of edges follows a binomial law, the multidegree centrality variance has the typical form $\sigma(L) = L_{max}q(1-q)$, with q the parameter of the random model.

1.7 Closed form of the distance for random multilayer and multiplex networks in the thermodynamic limit

Using Eqs.16, the layer multidegree centrality variance for random multilayer networks read:

$$\begin{aligned}\sigma^2(k_{\mathcal{Y}}) &= \sigma^2(2L_{max}^{intra} + L_{max}^{inter} + L_{max}^{replica}) \\ &= 4\sigma^2(L_{max}^{intra}) + \sigma^2(L_{max}^{inter}) + \sigma^2(L_{max}^{inter}) \\ &= N(N-1)(M-1)q(1-q)(1 + \frac{2}{M-1} + \frac{1}{N-1})\end{aligned} \tag{17}$$

Similarly $\sigma^2(k_{\mathcal{X}}) = M^2(N-1)q(1-q)(1+\frac{2}{N-1}+\frac{1}{M-1})$. Therefore, the nodewise standard deviation scales as $\sigma(k_{\mathcal{X}}) = M\sqrt{N}$ and the layerwise one scales as $\sigma(k_{\mathcal{Y}}) = N\sqrt{M}$ in the thermodynamic limit.

Using the previous equations and table we find that for random multilayer networks, the distances read:

$$d(k_{\mathcal{X}}, k_{\mathcal{X}}(r)) = r(1-p_{layer})NM\sqrt{q(1-q)} \tag{18}$$

$$d(k_{\mathcal{Y}}, k_{\mathcal{Y}}(r)) = r(1-p_{node})NM\sqrt{q(1-q)} \tag{19}$$

$$d_{\mathcal{Y}} = \frac{1-p_{node}}{1-p_{layer}} d_{\mathcal{X}}, \tag{20}$$

In the case of multiplex networks, distances as defined in Eq. 15 apply as well. For realistic purposes, we consider the case where the multiplex network has all its replica links that are not rewirable. i.e. the term $L_{max}^{replica} = 0$ (see previous table). Since we deal with multiplex networks, $L_{max}^{inter} = 0$ as well. Following the same canvas as in the previous part, we obtain $\sigma(k_{\mathcal{X}}) = \sqrt{M(N-1)q(1-q)}$ and $\sigma(k_{\mathcal{Y}}) = \sqrt{2N(N-1)q(1-q)}$. In the thermodynamical limit, the distances for random multiplex networks read:

$$d(k_{\mathcal{X}}, k_{\mathcal{X}}(r)) = r(1-p_{layer})N\sqrt{M}\sqrt{q(1-q)} \tag{21}$$

$$d(k_{\mathcal{Y}}, k_{\mathcal{Y}}(r)) = r(1-p_{node})N\sqrt{M}\sqrt{2q(1-q)} \tag{22}$$

$$d_{\mathcal{Y}} = \sqrt{2}\frac{1-p_{node}}{1-p_{layer}}d_{\mathcal{X}}, \tag{23}$$

The layerwise distance is $\sqrt{2}$ times higher than the nodewise one for the same condition as compared with random multilayer networks.

1.8 Distance for finite-size networks with power-law multidegree centrality distributions

In the case of infinitely large systems the standard deviations of networks exhibiting power-law multidegree centrality distributions might explode thus leading to diverging distances. However, in real systems power-law distributions can only extend up to a maximum value. By imposing a finite-cut-off on the largest multidegree one can therefore derive a closed-form expression of the distance for those systems, too. For example, let us consider a system whose node multidegree centrality distribution is $p(k_{\mathcal{X}}) = ak_{\mathcal{X}}^{-\gamma}$ with $k_{min\mathcal{X}}$ and $k_{max\mathcal{X}}$ being respectively the smallest and largest value of the actual multidegree centrality sequence. Here, we set $a = (\gamma-1)k_{min\mathcal{X}}^{\gamma-1}$ which corresponds to the normalization constant for a continuous power-law distribution [2].

Such a system can be physically obtained starting from a monolayer scale-free network with $p(k) = (\gamma-1)k^{\gamma-1}$ and randomly moving the links across the available M layers by keeping the connected nodes unchanged. This procedure gives a multiplex network whose node multidegree distribution $p(k_{\mathcal{X}}) = p(k)$ and whose layer multidegree distribution is binomial as a result of the randomization across layers. Because moment orders only depend on degree distributions, we can then use the well-known formula established for power-law configurations [3] to derive the node multidegree variance:

$$\sigma_{\mathcal{X}}^2 = \frac{(\gamma-1)k_{min\mathcal{X}}^{\gamma-1}}{3-\gamma}(k_{max\mathcal{X}}^{3-\gamma} - k_{min\mathcal{X}}^{3-\gamma}) - [\frac{(\gamma-1)k_{min\mathcal{X}}^{\gamma-1}}{2-\gamma}(k_{max\mathcal{X}}^{2-\gamma} - k_{min\mathcal{X}}^{2-\gamma})]^2 \tag{24}$$

This expression allows one to measure the node multidegree variance as the network size increases for different values of γ (**Fig T1**). Similar reasoning holds for systems with layer multidegree power-law distributions. Substituting the associated standard deviations in Eqs 2 of the main text one can eventually obtained the related nodewise and layerwise distances.

This result also allows to have an explicit expression of the dual relationship $d_Y(r) = c\frac{1-p_{node}}{1-p_{layer}}d_X(r)$, where $c = \frac{\sqrt{M}\sigma_{\mathcal{Y}}}{\sqrt{N}\sigma_{\mathcal{X}}}$ (Eq.3 in the main text). For example, in the case of the above introduced toy multiplex system with power-law node multidegree distribution and binomial layer multidegree distribution, the constant c has a more complex expression compared to random networks ($c = 1$), yet traceable:

$$c \approx \frac{N\sqrt{M}\sqrt{2q(1-q)}}{\sqrt{\frac{(\gamma-1)k_{min}^{\gamma-1}}{3-\gamma}(k_{max}^{3-\gamma} - k_{min}^{3-\gamma}) - [\frac{(\gamma-1)k_{min}^{\gamma-1}}{2-\gamma}(k_{max}^{2-\gamma} - k_{min}^{2-\gamma})]^2}} \tag{25}$$

1.9 Parameters for a complete uniform rewiring

The complete uniform rewiring reduces to find the probability parameters $p_{node}, p_{layer}, p_{tel}$ for which the effective probability for an edge to be rewired anywhere else is $\frac{1}{L_{max}-1}$ i.e. uniform across the network. Therefore, the probabilities depend on the size of the

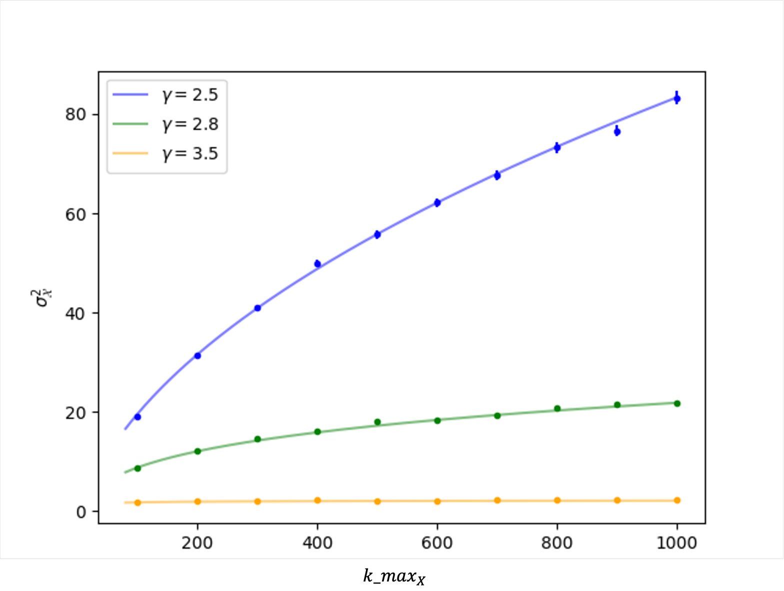

Fig T1. Variance of node multidegree centrality for finite-size networks with power-law distributions. Solid lines represent theoretical values from the previous equation. Points represent values obtained by averaging 10000 simulated networks with $M = 100$ and $N = k_{max\mathcal{X}} + 1$. Error bars account for standard error means. Note that by construction, results won't change if one chooses a different number of layers M.

system with $p_{node} = \frac{L_\lambda - 1}{L_{max} - 1}$ and $p_{layer} = \frac{L_p - 1}{L_{max} - 1}$ (see Table of section 1.4). The -1 term insures that we do not rewire the edge at its place.

For multilayer networks, the probability parameter for complete uniform rewiring read:

$$p_{node} = \frac{N^2 - 2}{(MN)^2 - 2} \tag{26}$$

$$p_{layer} = \frac{2(M^2 - 1)}{(MN)^2 - 2} \tag{27}$$

For $N >> 1, M >> 1$, $p_{node} \propto N^{-2}$ and $p_{layer} \propto M^{-2}$. Therefore, $p_{tel} = 1$ reduces to a complete uniform rewiring in the case of large multilayer networks.

For multiplex networks, the probability parameter for complete uniform rewiring read:

$$p_{node} = \frac{N(N-1) - 2}{MN(N-1) - 2} \tag{28}$$

$$p_{layer} = \frac{2(M - 1)}{MN(N-1) - 2} \tag{29}$$

For $N >> 1, M >> 1$, $p_{node} \propto N^{-2}$ and $p_{layer} \propto M^{-1}$. Therefore, $p_{tel} = 1$ reduces to a complete uniform rewiring in the case of large multiplex networks. With most real-world multiplex networks ($M \sim 1$), p_{layer} has often a non negligible contribution in the complete uniform rewiring.

1.10 Maximum of the distance for random multiplex networks in function of their size

We want to know where the closed-form of the distances for multiplex networks reach their maximum in function of the size N, M of the network. In general, the higher is N and M the higher are those distances. Since $d_{\mathcal{X}}$ and $d_{\mathcal{Y}}$ have the same N, M dependence, they reach their maximum for the same combination of N and M. Therefore, we use only $d_{\mathcal{X}}$ (Eq. 21 for the resolution). We set $a = N - M$, the variable and we fix $c = N + M$ as a constant. (see Fig. 3 in the main text). Eq. 21 read:

$$d_{\mathcal{X}} = r(1 - p_{layer}) \frac{(a+c)\sqrt{c-a}}{2\sqrt{2}} \tag{30}$$

It leads to:

$$\frac{\partial d_{\mathcal{X}}}{\partial a} = r(1 - p_{layer}) \frac{c - 3a}{2\sqrt{c-a}} \tag{31}$$

By setting $\frac{\partial d_{\mathcal{X}}}{\partial a} = 0$, it implies that the maximum of $d_{\mathcal{X}}$ is $a* = \frac{c}{3}$ leading to the position of the maximum when :

$$(N - M)^* = \frac{N + M}{3} \tag{32}$$

2 Real-world multiplex networks

We considered real-world multiplex networks gathered from disparate freely available datasets associated with published results. All multiplex networks were symmetrized and binarized before any analysis:

- **Twitter events**: 3 layers, from 88804 to 4377184 nodes. Nodes represent Twitter users, layers correspond to retweet, mentions and replies between users. Data are acquired througt the data API and in a time interval that corresponds to a specific event and filtered by keywords related to it [4, 5].

- **Uganda villages**: 2 layers, from 65 to 374 nodes. Nodes represent households bordering the Lake Victoria in Mayuge District, in Uganda and layers represent complete friendship and health advice between these households [6].

- **PierreAuger**: 16 layers, 514 nodes represent authors in the internal report repository of the Pierre Auger Collaboration. Edges represent coauthorship and layers are the category into which the reports fall [7].

- **Arxiv**: 13 layers, 14489 nodes. Nodes represent authors who posted an article on the preprint database Arxiv containing the word "networks" in the title or in the abstract up to May 2014. Edges represent coauthorship and layers are selected Arvix categories [7].

- **German transport**: from 35 to 279 layers, from 336 to 7565 nodes. Nodes represent stops (tram, subway, bus etc...), layers represent different lines and edges represent the connection between stops (intralayer) and the possible change between lines (interlayer) [8].

- **EuAir**: 37 layers, 450 nodes. Nodes represent european airports, layers represent 37 airlines company, edges, represent routes between airports [9].

- **Genetic**: 7 layers, from 367 to 18222 nodes. Nodes represent proteins, links represent protein-protein interactions. Layers represent interaction types between proteins [10, 11].

- **C.elegans**: 3 layers, 279 nodes. Nodes represent neurons of the nematode C.elegans, layers represent different synaptic junctions (electric, chemical monoadic, chemical polyadic), edges represent junctions between neurons [12, 13].
- **FAO**: 364 layers, 214 nodes. Nodes represent countries, layers represent food products, edges represent import/export relationships [11].
- **HumanMicrobiome**: 18 layers, 305 nodes. Nodes represent different eukaryotes population, and layers represent body sites [14, 15].

References

1. Newman M. Networks: An Introduction. OUP Oxford; 2010. Available from: `https://books.google.fr/books?id=LrFaU4XCsUoC`.
2. Barabàsi, Albert-László. Network science. Cambridge UK: Cambridge University Press; 2016.
3. Latora V, Nicosia V, Russo G. Complex networks: principles, methods and applications. Cambridge New York, NY Port Melbourne: Cambridge University Press; 2017.
4. Omodei E, De Domenico M, Arenas A. Characterizing interactions in online social networks during exceptional events. Frontiers in Physics. 2015;3.
5. De Domenico M, Altmann EG. Unraveling the Origin of Social Bursts in Collective Attention. Scientific Reports. 2020;10(1):4629. doi:10.1038/s41598-020-61523-z.
6. Chami GF, Ahnert SE, Kabatereine NB, Tukahebwa EM. Social network fragmentation and community health. Proceedings of the National Academy of Sciences. 2017;114(36). doi:10.1073/pnas.1700166114.
7. De Domenico M, Lancichinetti A, Arenas A, Rosvall M. Identifying Modular Flows on Multilayer Networks Reveals Highly Overlapping Organization in Interconnected Systems. Physical Review X. 2015;5(1):011027. doi:10.1103/PhysRevX.5.011027.
8. Bergermann K, Stoll M. Orientations and matrix function-based centralities in multiplex network analysis of urban public transport. Applied Network Science. 2021;6(1):90. doi:10.1007/s41109-021-00429-9.
9. Cardillo A, Gómez-Gardeñes J, Zanin M, Romance M, Papo D, Pozo Fd, et al. Emergence of network features from multiplexity. Scientific Reports. 2013;3(1):1344. doi:10.1038/srep01344.
10. Stark C. BioGRID: a general repository for interaction datasets. Nucleic Acids Research. 2006;34(90001):D535–D539. doi:10.1093/nar/gkj109.
11. De Domenico M, Nicosia V, Arenas A, Latora V. Structural reducibility of multilayer networks. Nature Communications. 2015;6(1):6864. doi:10.1038/ncomms7864.
12. Chen BL, Hall DH, Chklovskii DB. Wiring optimization can relate neuronal structure and function. Proceedings of the National Academy of Sciences. 2006;103(12):4723–4728. doi:10.1073/pnas.0506806103.
13. De Domenico M, Porter MA, Arenas A. MuxViz: a tool for multilayer analysis and visualization of networks. Journal of Complex Networks. 2015;3(2):159–176. doi:10.1093/comnet/cnu038.
14. Ding T, Schloss PD. Dynamics and associations of microbial community types across the human body. Nature. 2014;509(7500):357–360. doi:10.1038/nature13178.
15. De Domenico M, Biamonte J. Spectral Entropies as Information-Theoretic Tools for Complex Network Comparison. Physical Review X. 2016;6(4):041062. doi:10.1103/PhysRevX.6.041062.

Supplementary figures and tables

Node-layer duality in networked systems

Charley Presigny[1], Marie-Constance Corsi[1], Fabrizio De Vico Fallani[1,*]

1 Sorbonne Université, Institut du Cerveau - Paris Brain Institute - ICM, CNRS, Inria, Inserm, AP-HP, Hôpital de la Pitié Salpêtrière, F-75013, Paris, France

* corresponding author: fabrizio.de-vico-fallani@inria.fr

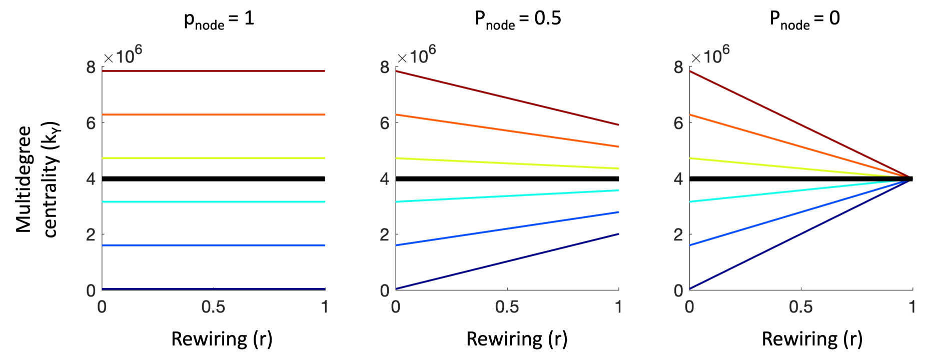

Fig S1. Effects of rewiring parameters on multidegree centrality. Values of layer multidegree centrality ($k^{\alpha}_{\mathcal{Y}}$) as a function of the amount of rewiring (r). Different colored lines correspond to selected layers with different initial multidegree centrality. Black lines denote the average layer multidegree centrality. Values are obtained from the theoretical formulas (Eqs 2 in the main text) for random networks with $N = M = 200$ and connection probability $q = 0.5$. The more the rewiring alters the connected layers (i.e. $p_{node} \to 0$ or equivalently $p_{layer} + p_{tel} \to 1$), the more their multidegree centrality will by attracted by the average value, leading to more homogeneous layerwise distributions. Same conclusions can be obtained for the node multidegree centrality $k^{i}_{\mathcal{X}}$.

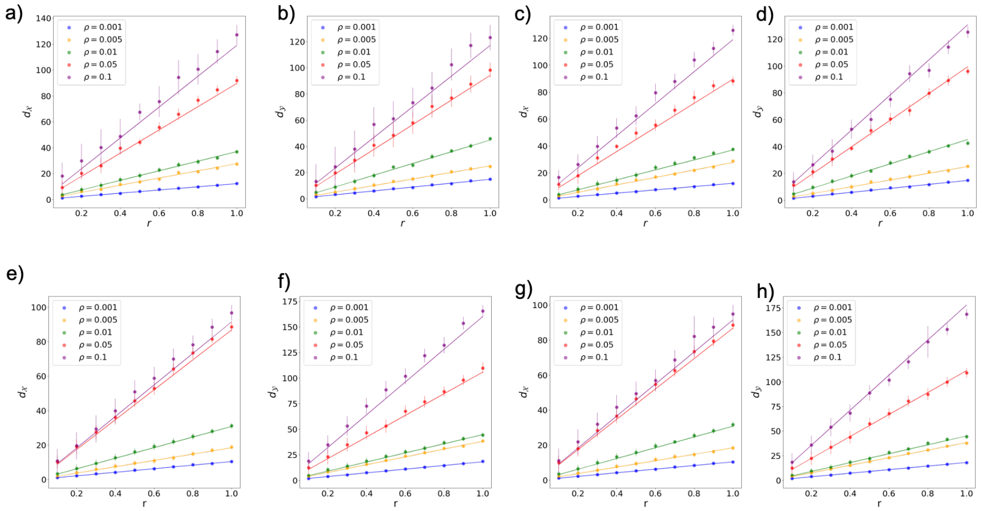

Fig S2. Comparison between analytical and numerical distances for different connection density values. Curves are the analytical distances as obtained with Eqs. 10,11 (and similar for the node multidegree centrality), Dots are the numerical distances. The numerical expected multidegree centrality is obtained by 100 rewiring of the initial multilayer/multiplex networks. Error represents 3 times the standard error of the mean. a) Nodewise distance as a function of r for random multilayer networks ($N = 20, M = 20$) at 5 different densities ρ ($p_{node} = 1$) b) Layerwise distance as a function of r for random multilayer networks ($N = 20, M = 20$) at 5 different densities ρ ($p_{layer} = 1$) c) Nodewise distance as a function of r for random multilayer networks ($N = 20, M = 20$) at 5 different densities ρ ($p_{tel} = 1$) d) Layerwise distance as a function of r for random multilayer networks ($N = 20, M = 20$) at 5 different densities ρ ($p_{tel} = 1$) e) Nodewise distance as a function of r for random multiplex networks ($N = 50, M = 50$) at 5 different densities ρ ($p_{node} = 1$) f) Layerwise distance as a function of r for random multiplex networks ($N = 50, M = 50$) at 5 different densities ρ ($p_{layer} = 1$) g) Nodewise distance as a function of r for random multiplex networks ($N = 20, M = 20$) at 5 different densities ρ ($p_{tel} = 1$) h) Layerwise distance as a function of r for random multiplex networks ($N = 20, M = 20$) at 5 different densities ρ ($p_{tel} = 1$).

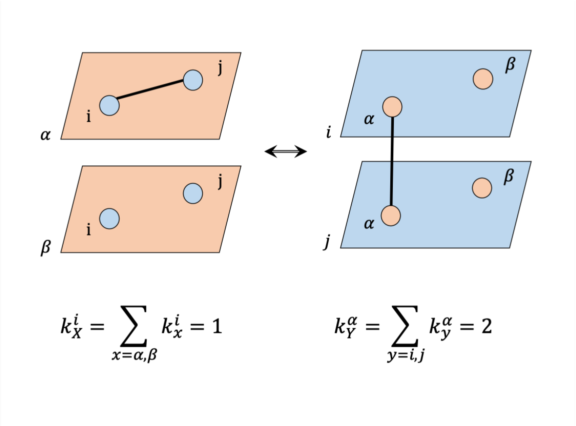

Fig S3. Effect of intralayer link additions on node and layer multidegree centralities. In general, adding one intralayer edge in the primal nodewise dimension increases the multidegree centrality in the dual layerwise dimension by a factor of 2 (right side), while the node multidegree centrality only increases by a factor of one (left side). Because in multiplex networks only changes within layers are allowed, this effect increments by construction the variance of the layer multidegree centrality and its distance as compared to the nodewise counterparts.

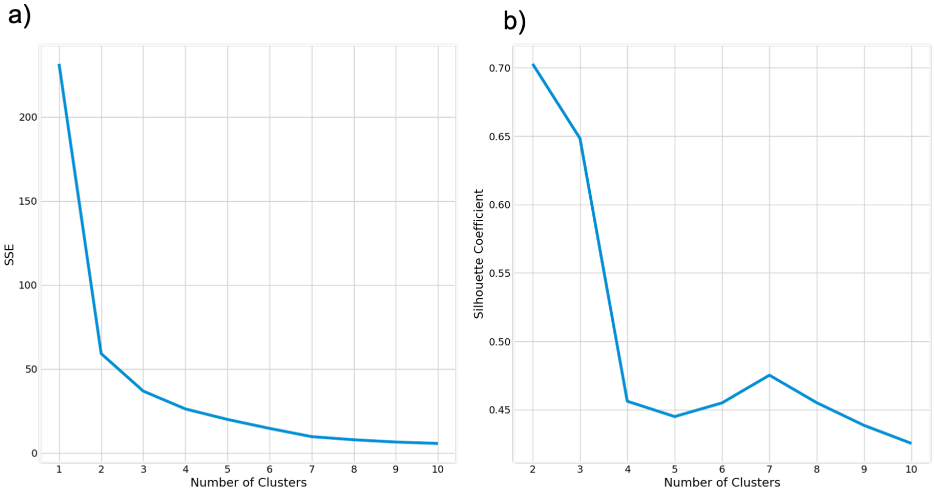

Fig S4. Optimal partition of the dual characterization of real-world multiplex networks (k-means clustering). a) Elbow method. Sum of the squared error in function of the number of clusters. The elbow point corresponds to the partition of space into two clusters which is considered as the optimum trade-off between error and the number of clusters (with knee-point detected using python package *kneed*). B) The silhouette coefficient in function of the number of clusters. The bigger is the silhouette coefficient, the more cohesive and separated the clusters. It values ranges from -1 to 1. Here the maximum is obtained for two clusters, $s = 0.70$.

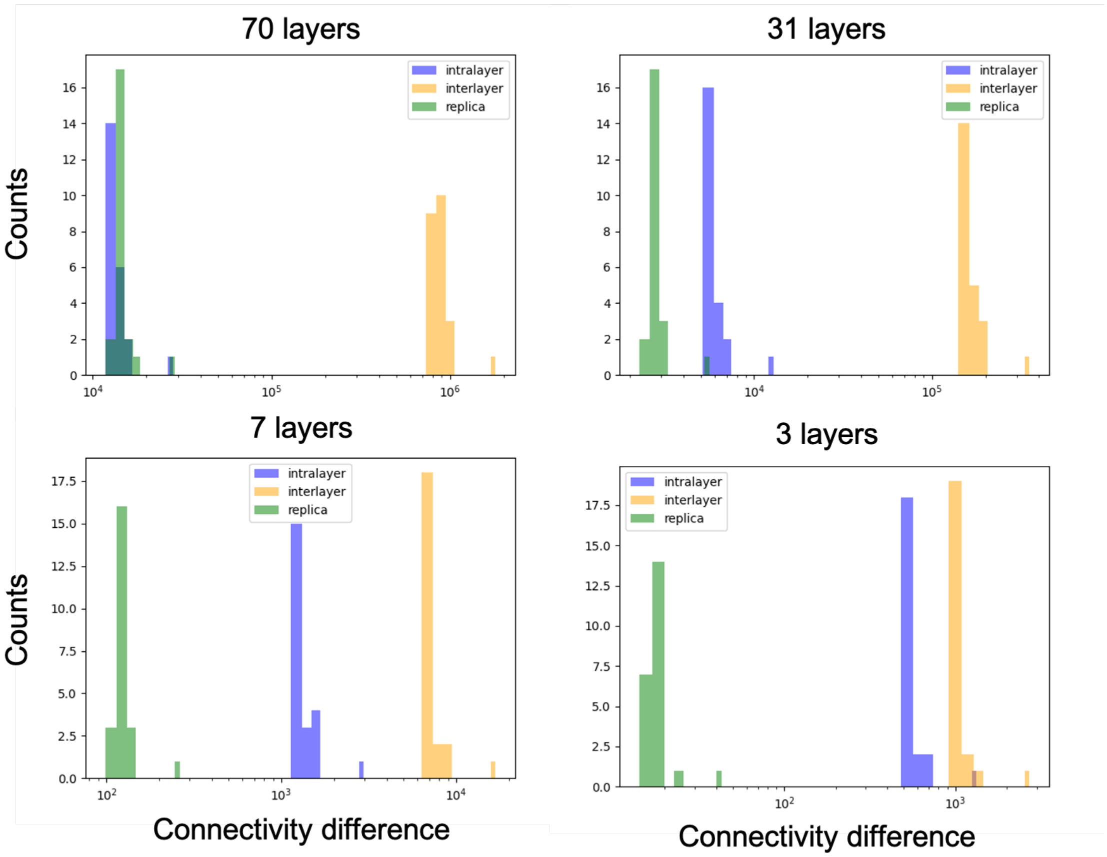

Fig S5. Distribution of connectivity changes in AD multifrequency brain networks across different number of layers. Connectivity changes are computed as the absolute value of the difference between the links' weights of each AD network and those from the group-averaged HC brain network. Colored histograms correspond to differences between replica (red), intralayer (blue) and interlayer links (yellow). The number of layers (frequencies) decreases from top-left to bottom-right panels. The number of nodes (brain areas) stay the same, i.e. $N = 70$.

Classes	$< \sigma_{\mathcal{X}} >$	$< \sigma_{\mathcal{Y}} >$
German Transport	21.05	141.5
Arxiv	13.67	8391
PierreAuger	29.42	2587
Uganda villages	12.20	363.2
Twitter events	82.02	1377000
HumanMicrobiome	39.98	357.9
C.Elegans	17.31	892.2
FAO trade	3312	1355
EuAir	27.12	203.2
Genetic	47.61	25150

Table S1. Average nodewise and layerwise standard deviations of the multidegree centrality for real-world multiplex networks.

ROI	Correlation	Original p-value	Corrected p-value
caudalanteriorcingulate L	0,53633	0,00833	0,02387
caudalanteriorcingulate R	0,51942	0,01108	0,02387
inferiorparietal R	0,46817	0,02426	0,02387
precentral R	0,45971	0,02732	0,02387
corpuscallosum R	0,45573	0,02886	0,02387
superiorfrontal R	0,45324	0,02985	0,02387
postcentral R	0,4418	0,0348	0,02387
superiorfrontal L	0,43931	0,03596	0,02387
supramarginal R	0,43832	0,03643	0,02387
paracentral L	0,43086	0,04012	0,02387
caudalmiddlefrontal R	0,42439	0,04355	0,02387
posteriorcingulate L	0,41344	0,04988	0,02387
superiorparietal R	0,4821	0,01983	0,04618
precuneus R	0,47364	0,02243	0,04618

Table S2. Spearman correlation between the AD patients' ROIs multidegree centrality and the MMSE clinical score. Only the most significant correlations after a cluster-based multiple correction are reported here ($p < 0.05$, last column). The primary cluster contains 12 ROIs, the secondary cluster contains only 2 ROIs.

.

Frequency	Spearman Correlation	Original p-value	Corrected p-value
9	0,60997	0,002	0,0374
8,5	0,60449	0,00225	0,0374
10,5	0,59454	0,00277	0,0374
9,5	0,59056	0,00301	0,0374
11,5	0,57613	0,00401	0,0374
10	0,56817	0,00468	0,0374
11	0,55772	0,00569	0,0374
8	0,55474	0,00601	0,0374
12	0,55126	0,0064	0,0374
7,5	0,49305	0,01683	0,0374
13,5	0,48708	0,01841	0,0374
7	0,47563	0,0218	0,0374
13	0,44777	0,03215	0,0374
14	0,43832	0,03643	0,0374
12,5	0,43036	0,04038	0,0374
14,5	0,42887	0,04116	0,0374
5,5	0,44827	0,03193	0,04902
3,5	0,44528	0,03323	0,04902
6	0,43334	0,03886	0,04902
5	0,42936	0,0409	0,04902
4	0,4219	0,04493	0,04902

Table S3. Spearman correlation between the AD patients' frequency multidegree centrality and the MMSE clinical score. Only the most significant correlations after a cluster-based multiple correction are reported here ($p < 0.05$, last column). The primary cluster contains 16 frequencies, the secondary cluster contains only 5 ROIs.
